# Supplementary material for: Silencing of Glycogen Synthase Kinase 3 Significantly Inhibits Chitin and Fatty Acid Metabolism in Asian Citrus Psyllid, Diaphorina citri
Source: Int J Mol Sci. 2022 Aug 25;23(17):9654. doi: 10.3390/ijms23179654 (PMC9455978; doi:10.3390/ijms23179654)
Supplement: Supplementary file 1 [file ijms-23-09654-s001.zip › Table S1.pdf]

Table S1. Primers used in this study

Primers	Sequences	Purpose
DcGSK3	F: ATGCAAATTATGAGACGGCT<br>R: CTAAGCCATGTCATTGGCTC	ORF amplify
DcGSK3	F: CCCCTCTGTTCAACTTCACCG<br>R: CGTGGTTTCCGAGAGGTCTGTAG	RT-qPCR analysis
GAPDH	F: CATGGCAAGTTCAACGGTGA<br>R: CGATGCCTTCTCAATGGTGG	
TPS1	F: GACATTTTCCGACTTTTCCCC<br>R: TTGCGGTCTACACGACAGCC	
Tre1-1	F: AAATCATCACCAAGGACAATCAA<br>R: GAAGAGCCACTTTTCAGCCAG	
Tre1-2	F: AAGCCAATGCTGGGAATCG<br>R: GGTCTGTGAAATCAGGGGGG	
Tre2	F: CAAGGAATGGTTGGAGGCTGT<br>R: GCGGAGATAGGTTACTGGGGT	
HK	F: GACTTTCTGGCGGCGTGTATC<br>R: TGGTTGTCTTGTCGTCTGTGGA	
G6PI	F: TGCTGTGGGACCAAGAGTTCAT<br>R: ACTTTGCCCGCATTTGTAGATAG	
GFAT	F: TGAGCATTCACAGACTCCGCA<br>R: GCGGATTTCGGGAATGTAGTC	
GNPNA	F: CGATTATGACCACGGGTTCCT<br>R: ACTTCACCAGAACGGCAATCA	
PAGM	F: GACAACTCAGGGGAAACTCAACT<br>R: GCAATACGGTCTCCATCCAACA	
UAP	F: AAGCCTGATGCTGTAGGAAATG<br>R: GCACAAGGAATGAATGACCAAT	
CHT	F: CGTGTATGCCTTTGCCGAT<br>R: TCCTTGAGTCTGACCAACCACC	
NAG	F: GTGGGAGTGGATGTGTTCGG<br>R: GCTTCAACTTGGACCAGAATAAAA	
ACC	F: CCTTCCCGTGTCCACTCCTTA<br>R: TACGAAGTTGAGGACCTGAGCA	
FAS	F: GAAGGTTATCCCCTCTCCCAAG<br>R: CAGGAGGTTGTTGGTGTGGTATT	
MCAD	F: CTGTTCAGGTGTTTGGAGGCA<br>R: GGGTTACTTTGGTATGTTGGGG	
GCD	F: GGACCAGATGCTCCTTCTTGCTA<br>R: AGTTGTGGGATTTGGTTGGC	
ACAT	F: CAGAAGGGAGTTGCCTCTATTTG<br>R: CGATAAACCAAAGTGTCCTCTCC	
Lipase	F: CCCAACAGGATTCCGTGACTAA<br>R: GAACGGTCACATTACCAATAGGG	

FAB	F: TCTGATTACTCGCAAGGTGGG	
	R: ACGCTCTTCACCTTACGACCG	
FAR2	F: ACGCTCTTCACCTTACGACCG	
	R: AATAGGACGAACACGGCAACC	
FAR1	F: GAACGCTTCCATAAAGACCCA	
	R: TTGACTCCTTCTTTTAGGGATTTG	
ELOVL7	F: CAGTCGTCAAAGTGGTTTTAGAGC	
	R: AGGGCAGTGATTTGATTGTTCTT	
ELOVL6	F: CTCTGAGGAAGGCTTTGATTGTC	
	R: AGGCATACCAGGTGTAGAGGAGC	
FA2H	F: TACATTGGGTATGGACGGGTTAG	
	R: GAGGAGAGTTGTCTGGAGGCTT	
Lipase 3	F: TACATTGGGTATGGACGGGTTAG	
	R: AGGAGAGTTGTCTGGAGGCTTC	
ELOVL1	F: TCCGCCTATTTACCGCCCT	
	R: GGTCCCGCATACAACACGAT	
Lipase 1	F: AGGCTATGATGTTTGGTTGTGG	
	R: TGTCGTAGACTCCCATTTCGTG	
LCFATP4	F: GAAGATGCTGGGTTTGAAGTGC	
	R: ACAAAGATTCCGACAGGGTGGT	
ALDH	F: CTCATCATCGGTGCTTGGAAC	
	R: ACAGAGGTAGAGCCCGTGTAGAA	
FAAH	F: CCACGGATGATGCTGAAACTG	
	R: ATCGGATGCCACTCCTACAATG	
ELOVL4	F: CAGTTCGCAATCATCCTATCCT	
	R: GTAGAAGTTGGCAAAGAGCAGTG	
LCFA4	F: CCCCGTGGTGAGATTCTGATT	
	R: ACCATCGCTTTCCTTCTTCGT	
LCFA6	F: GGAAAGGAGCCAAAGAATCAAA	
	R: CCAAGCCCAACAGACCAGAT	
ds*DcGSK3*	F: GGATCCTAATACGACTCACTATAGGATCATCAAAGTCCTCGGCA	dsRNA synthesis
	R: GGATCCTAATACGACTCACTATAGGGTTGAGATTCGGCTGGATG	
ds*GFP*	F: GGATCCTAATACGACTCACTATAGGCAGTGCTTCAGCCGCTACCC	
	R: GGATCCTAATACGACTCACTATAGGACTCCAGCAGGACCATGTGAT	

The underline indicates T7 promoter sequence.
